# Ross-Kabbani Operation in an Infant with Mitral Valve Dysplasia

**DOI:** 10.4061/2009/593659

**Published:** 2009-11-30

**Authors:** Carlo Pace Napoleone, Guido Oppido, Emanuela Angeli, Alessandro Giardini, Gaetano Gargiulo

**Affiliations:** Department of Pediatric Cardiac Surgery, University of Bologna Medical School, 40138 Bologna, Italy

## Abstract

*Background*. Mitral valve replacement can be very difficult to obtain in infants because the valve annulus diameter can be smaller than the available prosthesis. *Case Report*. We describe the case of a 2-month-old female weighing 3.5 kg affected by mitral valve dysplasia leading to severe valve stenosis. Despite full medication, the clinical conditions were critical and surgery was undertaken. The mitral valve was unsuitable for repair and the orifice of mitral anulus was 12 mm, too small for a mechanical prosthesis. Therefore, a Ross-Kabbani operation was undertaken, replacing the mitral valve with the pulmonary autograft and reconstructing the right ventricular outflow tract with an etherograft. *Results*. The postoperative course was uneventful and the clinical conditions are good at 4-month follow-up. *Conclusion*. The Ross-Kabbani operation can be an interesting alternative to mitral valve replacement in infants when valve repair is not achievable and there is little space for an intra-annular mechanical prosthesis implant.

## 1. Introduction

Congenital mitral valve dysplasia can become symptomatic at any age and sometimes may require surgical treatment very early in life. Mitral valve repair is not always achievable and the small size of the annulus can limit the possibility of mechanical prosthesis implant. In fact, the smallest available size of mechanical valves is 16 mm. A valve prosthesis of this size can be relatively difficult to place in also if some surgical “tricks”, like supra-annular implantation [1] can help the surgeon to allow this prosthesis to fit in the correct position. Furthermore, prostheses have numerous drawbacks in infants such as the lifelong anticoagulation and the need for reoperations for patient outgrowth. 

Hence, mitral valve replacement with pulmonary autograft (MVR-PA), the so-called Ross-Kabbani operation, may represent a suitable option in case of mitral dysplasia requiring valve replacement in infants [2]. 

We describe the case of a 2-month-old baby who underwent mitral valve substitution with pulmonary autograft.

## 2. Case Report

**Figure 1:**
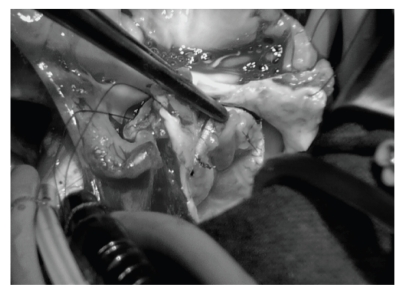
Intraoperative view showing the perfect continence of the autograft.

**Figure 2:**
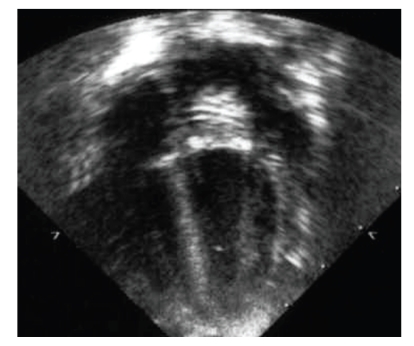
Postoperative echocardiografic aspect of the implanted autograft.

A 2-month-old female weighing 3.5 kg was referred to our centre with clinical signs of congestive heart failure. Shortly after birth, diagnosis of ventricular septal defect (VSD) was made. Due to failure to thrive, the baby underwent echocardiographic evaluation that revealed a mitral valve dysplasia compatible with hammock mitral valve with remarkable alteration of the subvalvular apparatus leading to severe stenosis. The mean gradient across the mitral valve was 20 mmHg. A 5 mm perimembranous VSD was also present. The clinical conditions were critical despite full medication and surgery was undertaken. A mild hypothermic cardiopulmonary bypass was established and direct inspection after trans-septal approach confirmed a very dysplastic valve absolutely unsuitable for repair. Perimembranous VSD was closed with bovine pericardial patch (Periguard; Bio-Vascular Inc., St. Paul, Minn, USA). The mitral valve was removed leaving a 12 mm diameter anulus incompatible with a intra-annular mechanical valve implant. The pulmonary valve was inspected and harvested with 3 mm infundibular muscular rim; the pulmonary trunk was sectioned just above the commissures. The pulmonary valve sized 10 mm and was mounted inside a 10 mm long woven Dacron tube graft of 12 mm of diameter (Vascutek, Renfrewshire, Scotland). The proximal and distal ends of the pulmonary autograft were then secured to the Dacron graft with three Prolene 7/0 running sutures respectively (Ethicon, Somerville, NJ, USA). The prosthesis was therefore doubly longitudinally incised to allow autograft grow and then implanted in the mitral anulus with 5/0 Prolene (Ethicon, Somerville, NJ, USA) interrupted stitches secured at the distal edge. A top-hat valve with pericardium, as recommended by Kabbani et al. [2], Ross and Kabbani [3], was not feasible for the small dimension of the left atrium. At the end of the procedure, valvular continence was optimal at saline injection (Figure 1). Right ventricular outflow tract was reconstructed with a 12 mm Contegra conduit (Medtronic Inc, Minneapolis, Minn, USA). At CPB suspension, persistent pulmonary hypertension was evident and inhaled NO therapy was started and switched to oral sildenafil 5 days later. The baby was discharged without complications and after 4 months, the echocardiogram showed trivial autograft gradient with no incompetence (Figure 2). 3D echocardiographic evaluation confirmed the good motion of the pulmonary autograft leaflets. 

## 3. Discussion

Mitral valve dysplasia is a wide spectrum of congenital morphologic abnormalities that affect the mitral valve resulting in stenosis, regurgitation, or both [4]. Despite conservative surgery always remains the first choice, some of these lesions can be very difficult to repair, and a valvular prosthesis must be implanted.

In infants, mitral valve replacement can be technically demanding and an ideal valve substitute is not available as yet. Bioprosthetic valves cannot be considered for their large size, the high stent profile, and the rapid calcification and degeneration in the younger. Small-size mitral homografts are not commonly available, difficult to implant, and not durable [5]. 

Mechanical prosthesis represents a valid option, even though the smallest size available, 16 mm, can be too large for intra-annular implant in the smaller infants. Moreover, high early and late mortality is reported after mitral prosthesis implant in patients less than one year of age [6]. In particular, estimated survival at 10 and 15 years follow-up was 33% ± 19% and 33% ± 27% in patients less then 2-year-old who underwent mitral valve replacement with mechanical prosthesis [7].

Therefore, the autologous pulmonary valve may represent a valid alternative in infants with small annulus to obviate the mismatch between the commercially available prosthesis and the anulus of the recipient, which has been reported to increase mortality [8]. 

Larger experience with Ross-Kabbani operation is present in literature among older patients. Kabbani and colleagues reported 80 patients with a mean age of 39.3 years treated by MVR-PA. Operative and late mortality were respectively 5.0% and 6.25%. At a mean follow-up of 25 months, no patients underwent reoperation and most of the surviving patients (83%) remain in class I status [2]. Authors stated that it is a two-valve operation and requires a “learning curve” and it may also be considered as the best alternative for infants and children with irreparable congenital mitral pathology [2].

Brown and colleagues reported 8 patients between 12 and 46 years of age who underwent MVR-PA. At a mean follow-up of 25.0 ± 6.2 months, 3 patients underwent pulmonary autograft replacement with mechanical valves. In two cases, progressive regurgitation was secondary to untreated systemic hypertension causing a prolapse of a single stretched leaflet of the pulmonary autograft. In the other patient, progressive stenosis was due to preservation of excessive native mitral leaflet and subannular tissue [9]. 

Moreover, avoidance of lifelong anticoagulation represents an invaluable benefit, especially in pediatric patients, in whom dosage management difficulties and side effects are particularly evident. In the cited series, oral anticoagulation therapy was assumed for 6 to 8 weeks, in case sinus rhythm was present [2, 9].

As far as grow potential is concerned, the Dacron conduit interruption is meant to preserve the possibility to grow of the autograft, but obviously longer follow-up is needed to evaluate the reoperation rate in this patients [10]. Anyway, we think that the possibility to implant a biological valve with the best diameter-effective flow ratio of an adequate dimension in a very small patient represents a valid option regardless of the autograft growth. 

There is very limited experience with the Ross Kabbani operation in infants and at the best of our knowledge 4 patients only, less than one year of age, have been reported in literature with a limited follow-up time [10–12]. 

## 4. Conclusions

The pulmonary autograft can be a valuable mitral valve substitute in infants when valve repair is not achievable and there is little space for an intra-annular mechanical prosthesis implant. Larger numbers and longer follow-up are indeed necessary to promote the pulmonary autograft as mitral valve substitute of choice in patients with mitral anulus smaller than 16 mm.
